# Advances in mRNA-Based Melanoma Vaccines: A Narrative Review of Lipid Nanoparticle and Dendritic Cell Delivery Platforms

**DOI:** 10.3390/cells15020099

**Published:** 2026-01-06

**Authors:** Connor K. Sisk, Laci M. Turner, Shafkat Meraj, Nabiha Yusuf

**Affiliations:** 1 Heersink School of Medicine, University of Alabama at Birmingham, 1670 University Blvd, Birmingham, AL 35294, USA; 2 Department of Dermatology, University of Alabama at Birmingham, 1670 University Blvd, VH566A, Birmingham, AL 35294, USA

**Keywords:** melanoma, mRNA vaccine, lipid nanoparticle, dendritic cell vaccines, combination therapy, neoantigen, personalized cancer vaccines, immunotherapy

## Abstract

**Highlights:**

**What are the main findings?**

- mRNA-based melanoma vaccines delivered through lipid nanoparticles (LNPs) or dendritic cells (DCs) generate strong neoantigen-specific T-cell responses.
- Personalized mRNA vaccine platforms show early clinical benefit, especially when combined with immune checkpoint inhibitors.

**What are the implications of the main findings?**

- Optimizing delivery systems and neoantigen selection may further enhance the therapeutic impact of mRNA melanoma vaccines.
- Hybrid and personalized vaccine strategies represent promising future directions for improving melanoma immunotherapy outcomes.

**Abstract:**

Melanoma remains one of the deadliest cutaneous malignancies worldwide, and despite advances in systemic therapy, recurrence and treatment resistance remain frequent challenges. Following the success of COVID-19 mRNA vaccines, mRNA-based cancer vaccines targeting melanoma antigens have emerged as a promising therapeutic direction. This review summarizes current evidence on mRNA melanoma vaccines, focusing on two leading delivery platforms: lipid nanoparticles (LNPs) and dendritic cell (DC) vaccines. A comprehensive search of MEDLINE, Embase, and Scopus from 2015 to 2025 identified clinical trials, preclinical studies, and review articles evaluating mRNA vaccine constructs and delivery strategies. Completed clinical studies demonstrate that personalized LNP-formulated mRNA vaccines can enhance neoantigen-specific T-cell responses and improve recurrence-free survival, particularly when combined with immune checkpoint inhibitors. DC-based mRNA vaccines also show potent immunogenicity, with stronger responses observed when DC maturation is optimized. Ongoing trials continue to investigate next-generation LNP formulations, DC priming strategies, and personalized neoantigen approaches. Overall, current evidence indicates that both LNP and DC platforms can augment antitumor immunity by broadening T-cell responses and enhancing checkpoint inhibition. Continued refinement of delivery vehicles, neoantigen selection, and scalable manufacturing processes will be essential to realizing the full clinical potential of mRNA vaccines in melanoma.

## 1. Introduction

Melanoma remains one of the most lethal cutaneous malignancies worldwide. While modern immunotherapies have transformed the therapeutic landscape, durable responses remain inconsistent, and many patients with advanced disease experience recurrence or treatment resistance. These limitations highlight a critical need for strategies that can generate consistent, robust antitumor immune memory. Advances in mRNA engineering, antigen discovery, and precision oncology have renewed interest in therapeutic cancer vaccines, particularly for melanoma.

Among emerging platforms, mRNA-based vaccines have gained substantial momentum due to their safety profile, rapid manufacturability, and capacity to encode highly individualized antigenic payloads. Two delivery vectors have emerged as leading candidates: lipid nanoparticles (LNPs), which protect mRNA transcripts and enhance uptake by antigen-presenting cells (APCs), and dendritic cell (DC)-based vaccines, which leverage the potent antigen-presenting capabilities of ex vivo-engineered DCs. This review examines the mechanistic foundations, advantages, and limitations of mRNA vaccines in melanoma, focusing on LNP and DC-based delivery systems, their investigational landscapes, and future directions shaping their therapeutic potential.

A comprehensive literature search was conducted across Embase, MEDLINE (via PubMed), and Scopus to identify studies evaluating mRNA-based vaccines, mRNA immunotherapies, and RNA-based melanoma treatments. The search included records published from 2015 to October 2025 and was limited to articles available in English. Core search terms used across databases included “mRNA vaccine” AND “melanoma,” “mRNA therapeutics” AND “immune response,” and “melanoma neoantigen vaccine” AND “RNA.” Detailed search strategies for Scopus, MEDLINE, and Embase are available upon request.

The initial search identified 533 records from Embase, 260 from MEDLINE, and 556 from Scopus, yielding a total of 1349 records. After the removal of 652 duplicate records, 697 unique articles remained for screening. All references were imported into Covidence, a systematic review management platform used for title/abstract screening, full-text review, data extraction, and study organization. Two reviewers independently screened titles and abstracts, with a third reviewer resolving conflicts. Following this stage, 141 studies met the criteria for full-text review. Full-text screening was performed by two additional reviewers, and after applying all inclusion and exclusion criteria, 79 studies were selected for qualitative synthesis and data extraction for this narrative review.

Inclusion criteria included recent review articles (2015–2025) relevant to mRNA vaccines or melanoma immunotherapy, phase I–III clinical trials (ClinicalTrials.gov) involving mRNA vaccines or RNA-based immunotherapies, original research articles published within the past 10 years, and articles available in English with full-text accessibility. Exclusion criteria included articles without full-text availability, conference abstracts, posters, or presentations, non-peer-reviewed sources, and publications not focused on mRNA therapeutics or melanoma.

## 2. Melanoma and Conventional Treatment Modalities

### 2.1. Disease Overview

Cutaneous melanoma remains the most lethal form of skin cancer despite representing 1.7% of cutaneous malignancies [1,2]. Melanoma accounts for an estimated 325,000 new diagnoses made and 57,000 deaths per year worldwide, representing over 80% of skin cancer-related mortality [1]. Global incidence of melanoma has risen in recent years, increasing from about 20 new cases per 100,000 in 2012 to 23.5 new cases per 100,000 in 2022, with the highest rates observed in men approaching 30 new cases per 100,000 in 2022 [2]. These epidemiologic trends emphasize the urgency for therapeutic advancement.

Melanoma risk is shaped by a mix of genetic traits, pigment-related features and patterns of behaviors of ultraviolet (UV) radiation exposure. Melanocortin-1-receptor (MC1R) gene variants influence skin phototype, and people with red hair, fair skin, or low eumelanin levels are more sensitive to UV damage and therefore at higher risk of developing melanoma [3]. A greater number of acquired nevi, the red-hair phenotype, and MC1R R alleles each independently increase melanoma susceptibility, with one study showing a 25-fold higher risk in individuals with ≥20 nevi and the MC1R R/R genotype [3]. UV exposure remains the most important modifiable risk factor. Intermittent, intense sun exposure (UV-B) during childhood or adolescence, especially more than five blistering sunburns, nearly doubles melanoma risk [4]. Both UV-B and UV-A contribute to carcinogenesis, and tanning beds (UV-A) are particularly dangerous, increasing melanoma risk by 75% in users under age 35 [5]. 

Family history of melanoma is another important risk factor, with 5–10% of cases showing familial patterns, highlighting the role of heritable genetic factors [6]. Certain high-risk syndromes, such as familial atypical multiple mole and melanoma syndrome (FAMMM) or dysplastic nevus syndrome (DNS), are characterized by numerous atypical nevi and carry an extremely high lifetime risk of melanoma [6]. Even outside of these syndromes, people with a large number of nevi have a significantly increased risk, and observational studies consistently support the strong link between nevus burden and melanoma development [7]. Individuals with a personal history of melanoma are also more likely to develop additional primary melanomas [8]. Consequently, patients with many nevi, dysplastic lesions, or a strong family history are typically followed regularly every 3 to 6 months.

Most melanomas develop on skin that experiences intermittent sun exposure and burns easily, and people with repeated intense sun exposure carry the highest risk. This is underscored by the fact that patients who intentionally reduce UV exposure after a melanoma diagnosis have a lower likelihood of developing a second primary melanoma [9]. In contrast, individuals with darker skin or those who tan easily without burning have substantially lower melanoma rates, although melanoma can still occur in areas not chronically exposed to the sun [10]. The age at which UV exposure occurs matters as well: intermittent, intense sun exposure in childhood or adolescence, especially more than five blistering sunburns, significantly increases melanoma risk [11].

### 2.2. Pathophysiology and Immune Response

The tumorigenesis of melanoma is driven by a multistep process of genetic and immunologic dysfunction. UV accumulated from the environment promotes DNA damage within cutaneous melanocytes, driving mutagenic disease processes [2]. Primarily, gain-of-function mutations in key oncogenes, such as *BRAF* (most commonly *BRAFV600E*), *NRAS*, *GNAQ*, *GNA11*, and *KIT*, collectively enhance mitogen-activated protein kinase (MAPK) pathway signaling and confer the proliferative changes necessary for early malignant transformation [1,2]. At this stage of tumorigenesis, cellular senescence mechanisms may still intervene to halt the pathologic growth. However, subsequent loss-of-function mutations in tumor suppressor genes, such as *CDKN2A*, *TP53*, *PTEN*, *BAP1*, and *NF1*, allow for escape from cellular senescence and progression from benign nevi onward to intermediate lesions to advanced, potentially invasive melanoma [1].

Melanization adds complexity to melanoma biology and can influence both prognosis and tumor behavior. Melanization, also called melanogenesis, is the process of melanin production by melanocytes. The relationship between melanization and melanoma tumor biology is complex and suspected to be stage-dependent [12]. Relating to melanoma, melanin is protective against UV-induced damage and resulting carcinogenesis but has also been associated with some toxicities in melanoma [12]. In early disease stages, strongly pigmented tumors have shown better prognosis and tumor survival (91.4%) compared to amelanotic tumors (67.7%), which are also associated with worse prognosis and higher risk of metastasis [13,14]. However, in advanced disease, increased melanin is associated with decreased radiotherapy efficacy and poorer post-treatment survival compared to amelanotic tumors [15]. This paradoxical loss of the protective effect of melanin in later disease states is thought to be due to the free radical scavenging and radioprotective characteristics of melanin, shielding tumor cells from radiotherapy [15]. 

Studies have also shown stage-dependent pigmentation changes. For example, less melanization has been associated with deep primary tumors, while greater pigmentation is more commonly observed in metastatic tumors [15]. At the molecular level, active melanogenesis produces mutagenic and cytotoxic intermediates that enhance tumor progression and treatment resistance [12]. Varying levels of melanization may also influence how “visible” melanoma cells are to the host’s immune system [12]. Altered melanogenesis can influence antigen presentation, T-cell signaling, and the tumor microenvironment (TME) [12]. Further understanding of the molecular role of pigmentation in melanoma pathogenesis and identification of related antigen targets may provide novel avenues for modern cancer therapeutics. 

Melanoma-derived melanosomes can also be transferred indirectly to antigen-presenting cells (APCs), where they can influence immune response. However, dendritic cell maturation appears to be driven primarily by the apoptotic states of melanoma cells, rather than by melanin content alone. Although amelanotic melanomas have decreased expression of melanocyte differentiation antigens regulated by microphthalmia-associated transcription factor (MITF), they show comparable tumor-infiltrating lymphocyte quantities to pigmented melanomas, even when adjusted for tumor thickness [16,17]. This finding suggests that even though amelanotic melanomas express fewer pigmentation-related antigens, they can still be recognized by the immune system to a similar extent as melanotic melanomas when at similar tumor stages. Clinically, amelanotic melanomas may evade early immune detection, not due to impaired dendritic cell function, but due to reduced expression of melanocyte differentiation antigens that are immunotherapy targets, along with a more subtle clinical presentation that may complicate or delay diagnosis [16,17,18]. 

The malignant progression of melanoma can be exacerbated by disturbances to normal antitumor immunity. Recognition of tumor cells by the adaptive immune system can be impaired by loss of tumor antigenicity, failures in tumor antigen processing, presentation, and recognition, and interference with normal antitumor cytokine signaling [1]. Consequently, a robust CD8^+^ T-cell adaptive response and sufficient infiltration of cytotoxic lymphocytes may not occur, resulting in an immunologically “cold” TME that is poorly responsive to both the natural immune response as well as contemporary immunotherapies [19]. Thus, therapeutic approaches to overcoming this hypothermic, evasive TME fundamentally derive from efforts to enhance antigen presentation, restore T-cell activation, and convert “cold” tumors into inflamed, treatment-responsive TMEs [20].

### 2.3. Conventional Melanoma Therapeutics

Therapeutic management of melanoma has evolved substantially over the past two decades, although durable resolution of advanced disease remains a challenge. Early systemic therapies, such as interferon-γ (IFN-γ) and high-dose interleukin-2 (IL-2), were among the first medications used to facilitate substantial antitumor responses. However, their clinical utility was limited by severe adverse events (AEs), including cytokine storm, capillary-leak physiology, and multiorgan dysfunction. The discovery of melanoma driver mutations enabled the development of targeted therapies like BRAF inhibitors (e.g., vemurafenib, dabrafenib) and MEK inhibitors (e.g., trametinib, cobimetinib), which have been shown to induce rapid, although transient, tumor regression due to tumor resistance mechanisms [21].

Immune checkpoint inhibitors (ICIs) provided a revolutionary addition to melanoma therapeutics through manipulation of the endogenous T-cell response. Cytotoxic T-lymphocyte-associated protein 4 (CTLA-4) blockade with ipilimumab stimulates early T-cell priming, bolstering the adaptive tumor response and improving overall survival, although response rates are modest [21,22]. Programmed death-1 (PD-1) inhibitors (e.g., nivolumab, pembrolizumab) and programmed death-ligand 1 (PD-L1) inhibitors (e.g., atezolizumab) have demonstrated efficacy in preventing PD-1/PD-L1-mediated T-cell exhaustion and sustaining cytotoxic activity in the TME [21]. However, response to checkpoint blockade is insufficient: approximately 50% of patients with metastatic melanoma are non-responders, and among patients treated adjuvantly post-resection, nearly half experience disease recurrence [22]. Proposed mechanisms of ICI resistance largely implicate previously mentioned means of immune surveillance evasion and derive from the understanding that ICIs amplify existing immune responses rather than improving de novo tumor antigen recognition and initiation of adaptive immunity [2].

Additional therapeutic modalities offer clinical benefit in specific contexts. For example, oncolytic viral therapy (T-VEC), a genetically modified HSV-1 virus engineered for selective tumor lysis and enhanced release of tumor antigens, is approved for injectable, unresectable cutaneous melanoma. Utilizing T-VEC, the OPTiM trial (Phase III) demonstrated an improved response rate versus control (16.3% vs. 2.1%) [23]. Engineered IL-2 variants, such as nemvaleukin alfa, confer IL-2-mediated antitumor effects with reduced toxicity and show promise, particularly within combination therapy approaches, but require further long-term validation. Topical imiquimod and isolated limb perfusion therapies have situational utility but comparatively narrow roles in modern practice [21].

Although earlier detection and advances in systemic therapies have improved outcomes, prognosis remains poor for patients with advanced or high-stage disease. Five-year survival rates for Stage III and Stage IV melanoma have been estimated at roughly 60% and 16%, respectively [24]. In this context, cancer vaccines, particularly mRNA-based platforms similar to those implicated in the Pfizer/BioNTech and Moderna COVID-19 vaccines, have emerged as a promising strategy for augmenting antitumor immunity and improving outcomes in melanoma patients.

## 3. mRNA Vaccine Overview

### 3.1. Immunological Mechanisms of mRNA Vaccines

mRNA vaccines leverage the host’s own cellular machinery to generate tumor antigens, enabling a controlled simulation of natural infection that triggers a potent adaptive immune response. In melanoma, a highly mutagenic malignancy, this strategy is particularly advantageous, as the abundance of non-self neoantigens facilitates efficient T-cell priming once they are appropriately presented by APCs. The promising potential of mRNA vaccines derives from their ability to both broaden and intensify antigen presentation while avoiding many of the tolerance mechanisms that limit conventional peptide- or protein-based vaccines.

Current mRNA vaccine platforms generally fall into two categories: personalized neoantigen vaccines and more generalized “off-the-shelf” vaccines [25]. Personalized neoantigen vaccines rely on patient-specific mutational profiles to generate highly individualized targets [24]. Their specificity to an individual patient’s unique tumor signature allows for circumventing tolerance to self-antigens, eliciting more selective cytotoxic T-cell activity, and reducing off-target AEs [26]. In contrast, “off-the-shelf” vaccines incorporate broadly shared tumor antigens, such as tumor-specific antigens (TSAs) and tumor-associated antigens (TAAs), or lineage-restricted proteins [24,25]. In place of patient specificity, this modality offers broader scalability but also may provoke weaker T-cell responses and exhibit diminished efficacy due to central immune tolerance and the potential for autoimmune toxicity [25,27].

Once delivered, the mRNA construct acts as a template for intracellular translation of the encoded antigen using host machinery. These antigenic proteins undergo cytosolic processing and are subsequently displayed on MHC class I and MHC class II complexes, enabling coordinated activation of CD8^+^ cytotoxic T lymphocytes and CD4^+^ helper T cells, respectively [24]. This dual presentation is central to generating durable antitumor immunity: CD8^+^ T cells mediate direct tumor cell killing, while CD4^+^ T cells enhance DC licensing, promote cytokine production, and support the maintenance of memory T-cell populations [24]. In addition to antigen expression, mRNA constructs may be engineered to encode immune-stimulatory cytokines, co-stimulatory molecules, or adjuvant-like motifs that further augment antigen presentation and promote Th1-skewed immune activation [28]. This process promotes a coordinated cellular immune response against cancer cells expressing the same introduced antigen, enhancing tumor visibility and overcoming some of the key mechanisms of immune evasion that limit other therapeutic modalities [24].

Efficient delivery is achieved through formulation of the mRNA within protective vectors, most commonly LNPs, which shield transcripts from extracellular degradation and facilitate uptake by APCs. Following endocytosis and cytosolic release, translated protein becomes accessible to the host antigen-processing machinery. The resulting expansion of antigen-specific effector T cells directly counteracts several mechanisms of melanoma immune evasion, including impaired antigen presentation, T-cell dysfunction, and the development of immunologically “cold” tumor microenvironments [19,24].

Modern mRNA vaccine platforms incorporate a variety of biochemical and structural modifications to improve the stability, translational efficiency, and immunogenic potency of delivered constructs. Enzymatic capping strategies and synthetic cap analogs enhance ribosomal recognition and prevent innate immune degradatory processes. Additional refinements, such as optimizing the length of the poly(A) tail and codons, increasing the G:C content, incorporating nucleosides, and optimizing mRNA purity through fast protein liquid chromatography or high-performance liquid chromatography purification methods, improve transcript durability and protein yield [28].

### 3.2. Advantages of mRNA Vaccines

mRNA vaccines offer several advantages over traditional vaccine and immunotherapy platforms. Firstly, mRNA constructs do not require integration into the host genome, giving them a better inherent safety profile than DNA- or viral vector-based vaccines. Further, their transient expression reduces long-term safety concerns while still permitting induction of a robust and adaptive immune response, although some highly rare instances of mRNA vaccine integration into host cells have been observed [29].

A major population-level benefit of mRNA technology is its rapid and scalable manufacturability. Once a given tumor is mutationally characterized, individualized mRNA vaccine sequences can be generated and synthesized with rapid turnaround [29]. Additionally, the absence of complex protein purification processes, combined with cell-free synthesis methodologies, supports cost-efficient production and scalability. This characteristic enables a level of personalization that is not yet feasible with other platforms, especially protein-based vaccines. 

Personalization represents another key advantage of mRNA vaccine technology [12]. Broadly, patient- and/or tumor-specific neoantigens can be identified and compiled into individualized tumor mutation profiles [24]. Subsequently, a single mRNA vaccine can be synthesized with a sequence encompassing each of the selected neoantigen targets. In theory, this specificity significantly enhances both the targeting and efficacy of pre-existing and de novo CD4^+^ and CD8^+^ T-cell antitumor responses [12,30]. This highly personalizable technology may enable focal targeted immunogenicity with minimized off-target immune activation and toxicity. 

mRNA vaccines exhibit strong intrinsic immunogenicity even in the absence of highly antigenic tumors. By encoding tumor-specific neoantigens, mRNA vaccines can effectively supply the immune system with a diverse range of highly specific targets to “try” [21]. This benefit proves particularly advantageous in settings where tumors have downregulated antigen presentation or evolved mechanisms that might otherwise limit engagement with T cells [31]. Moreover, the ability to co-encode immune-stimulatory molecules or adjuvants enables simultaneous enhancement of APC activation and promotion of favorable Th1-skewed responses without requiring an additional therapeutic [28].

### 3.3. Challenges of mRNA Vaccines

mRNA vaccines face several fundamental challenges that limit their effectiveness without appropriate delivery systems. Unprotected or “naked” mRNA is inherently unstable [32]. Extracellular RNases facilitate rapid mRNA degradation, leading to an inability to achieve optimal intracellular translation [33]. In addition, the large size and pronounced negative charge of mRNA impairs passive diffusion across cellular membranes, resulting in inefficient uptake into APCs [32].

Naked mRNA also triggers innate immune pathways that restrict its overall utility as a therapeutic. Recognition by toll-like receptors (TLRs), such as TLR3, TLR7, TLR8, and RIG-I-like receptors, can induce type I interferon activity, which limits translational efficiency and generates unwanted inflammation [34]. In turn, optimized delivery vectors are important for stabilizing mRNA to prevent unwanted inflammation and optimize vaccine performance.

These constraints translate into suboptimal clinical activity. In a phase I melanoma trial (NCT03394937) evaluating naked mRNA, immune responses were observed in only 33% of patients in the high-dose cohort [35]. This finding was attributed primarily to extracellular degradation and insufficient cellular uptake rather than deficiencies in antigen design [35]. Similar limitations have led to restricted use of naked mRNA across oncology trials, prompting a shift toward biologic and synthetic carrier systems capable of stabilizing transcripts, enhancing delivery to DCs, and improving cytosolic release [33]. 

Safety and AEs additionally represent an important consideration with mRNA vaccines. In general, mRNA vaccines are associated with self-limited AEs, including injection-site pain, headache, fever, fatigue, and myalgia [36]. Some rare side effects, such as myocarditis, lymphadenopathy, and anaphylaxis, have been reported in various instances of mRNA vaccine administration, nonspecific to melanoma [36]. In melanoma-specific mRNA vaccine trials, vaccines have generally been reported as being well tolerated, with the most commonly reported side effects limited to injection-site reaction, chills, and fatigue [37]. These side effects are typically short-term and show decreased frequency or severity with subsequent dosing [38]. Serious AEs have been uncommon in clinical studies, further suggesting the overall favorable safety profile of this technology.

Collectively, these challenges underscore the need for optimized delivery vectors, such as LNPs and transfected DCs, to overcome the intrinsic instability and immunologic barriers associated with naked mRNA and enable reliable antitumor immunogenicity.

## 4. Types of mRNA Vaccines

### 4.1. Dendritic Cell-Based mRNA Vaccines Overview and Vaccine Mechanism

Recent data from in vitro and in vivo studies on the application of DCs as antitumor vaccines, specifically targeting melanoma, have shown great promise. DCs are critical in vaccine development, as they are cells of the innate immune system and potent professional APCs that connect the innate immune system with the adaptive immune system. Most DCs in the body remain in an immature state until activated by the necessary specific stimuli [39]. When activated, DCs initiate adaptive immune responses by serving as key mediators in B-Cell and T-Cell priming, activation, and immunity, allowing for the activation of primary and secondary effector immunologic pathways [40]. DCs play a central role in activating CD4^+^ helper T cells, CD8^+^ cytotoxic T lymphocytes (CTLs), and natural killer cells (NKs). As professional antigen-presenting cells, DCs process tumor-derived antigens upon encountering tumor cells and display them on peptide–MHC I and MHC II complexes. This presentation provides T cells with the essential first activation signal, guiding their differentiation into CD8^+^ CTLs and CD4^+^ helper T cells, respectively [40]. Once stimulated by DCs, Th1 cells secrete IFN-γ and tumor necrosis factor-alpha (TNF-α), both of which contribute to antitumor activity and cell death by activating the signaling pathways of nuclear factor-κB (NF-κB), MAPK, and JAK/STAT [41]. 

DCs also display critical co-stimulatory molecules such as CD80 and CD86 on their surface, which are also needed to supply the essential secondary signals needed to activate and differentiate T cells, and with this stimulation, naïve T cells go on to differentiate into specialized CTLs and induce targeted antitumor effects [42]. In addition, CD4^+^ T-helper cells produce IL-2, a key cytokine that drives T-cell proliferation, promotes CTL activation, and enhances the function of natural killer cells and CD8^+^ CTLs [40]. Lastly, DCs are also key immunoregulators, as they express co-stimulatory molecules such as CD70 and OX40L for CD8+ effector function and memory formation, and they secrete cytokines, including IL-12 and type I interferons, to regulate T-cell priming and survival [43].

DC-based mRNA vaccines attempt to leverage these natural immune-activating abilities of DCs. When equipped with the mRNA that can encode tumor-associated antigens, the DCs translate this mRNA into antigenic proteins that are then presented as antigens to the body’s immune system. Upon doing so, DCs and the vaccine can activate CTLs and recruit NK cells for tumor growth inhibition and elimination. Simultaneously, these DCs also activate naïve B cells and T follicular helper (Tfh) cells, and the activated B cells enter germinal centers, where they undergo clonal selection and affinity maturation to produce high-affinity antibodies with high antigen-specificity [40]. Additionally, an advantage of DC-based mRNA vaccines is due to the ability of DCs to migrate from the site of antigen uptake to sites of lymphoid organs where immune responses are initiated, and this migratory capacity of DCs allows for efficient activation, proliferation, and differentiation of naïve T cells and coordinated interactions with B cells and T follicular helper cells for B-cell and antibody-mediated memory formation [44]. This combination of humoral and cell-mediated immune responses by the vaccine DCs helps generate immediate immunity and long-lasting memory generation for more durable and targeted immunity against melanoma and other cancers.

#### 4.1.1. Vaccine Preparation and mRNA Loading Techniques

DCs are advantageous for mRNA vaccines due to their ability to elicit specific T-cell-mediated immune responses in response to cancer antigens [45]. For vaccine preparation, DC precursors are isolated from either CD34+ precursor cells from the bone marrow or from the peripheral blood mononuclear cell population from patients [46]. Then, these immature DCs are grown in lab settings with various growth factors to generate mature DCs ready to be loaded with mRNA for vaccine delivery. Granulocyte-macrophage colony-stimulating factor (GM-CSF) and IL-4 are seen as the major growth factors necessary for DC maturation and differentiation [47]. 

Upon DC maturation, DCs are loaded and pulsed with mRNA and tumor antigens. Historically, some of the earliest trials and studies have employed several methods for loading antigens into DC vaccines, including the direct use of antigenic peptides and isolated native peptides, pulsating DCs with recombinant proteins, tumor lysates, and DC-tumor cell hybrids [48]. With the emergence of mRNA-based cancer immunotherapy, DCs have become ideal targets for both in vivo and ex vivo mRNA delivery [43]. DCs, particularly monocyte-derived DCs, can be efficiently transfected ex vivo with mRNA, and electroporation of these cells with the intended tumor antigen mRNA has become a preferred loading strategy because it achieves high transfection efficiency without requiring carrier molecules [45]. Other recent techniques include using co-culture, cell fusion, and transfection methods to pulse the immature DCs with tumor antigens in the form of nucleic acids encoding TAA ex vivo, apoptotic tumor cells, live tumor cells, and viral vectors [46]. Studies evaluating the efficacy and antitumor effects of DC-based mRNA vaccines analyze DC maturation markers CD80, CD86, CD83, and MHC I when inside the body, with higher levels of maturation markers correlated with greater immunogenicity and effective vaccine response [46].

#### 4.1.2. Limitations of Dendritic Cell-Based mRNA Vaccines

While DC-based mRNA vaccines provide great potential for immunotherapy, existing limitations and challenges persist. Human DCs can be divided into a few major groups, including Plasmacytoid DC (pDC), Conventional DC (cDC), Monocyte-derived DCs (MoDC) subsets, migratory DCs, and Langerhans cells [42,49]. Among these, pDCs can serve as a barrier to vaccine development and immune protection, as they can lessen the antitumor activity of both NK cells and T cells by overexpressing PD-L1, a transmembrane immune response inhibitor that tumor cells frequently use to evade tumor death and apoptosis. Similarly, pDC-derived IL-10 is seen as a major influence on the immunosuppressive tumor microenvironment, as it can lead to low MHC I expression and reduced subsequent CTL activation, making it difficult for DCs to present tumor antigens and mount an effective and robust anti-cancer response, particularly against melanoma [42,49]. Additionally, the tumor immunosuppressive microenvironment limits the clinical efficacy of DC vaccines due to the presence of suppressive myeloid cells and inhibitory molecules and the infiltration of regulatory T cells (Tregs) [50]. Completed and ongoing preclinical and clinical trials have attempted to create DC-based mRNA vaccines to mitigate some of these adverse interactions of DCs with the tumor microenvironment.

Off-target risks such as target genes integrating with DC genomes also pose risks to clinical efficacy, while DCs’ adverse interactions with the immunosuppressive tumor microenvironment make it difficult to mount durable and effective antitumor responses [33,42]. To optimize DC vaccination and its intended T-cell responses against melanoma, uncovering and fixing issues surrounding preparation, mRNA transfection and DC loading, what types of adjuvants, and DC activation and maturation when interacting with the tumor cells are necessary [51].

### 4.2. Lipid Nanoparticle mRNA Vaccines

#### 4.2.1. Lipid Nanoparticle mRNA Vaccine Mechanism and Structure

LNPs have emerged as one of the leading non-viral delivery platforms for mRNA cancer vaccines and are increasingly being investigated for application in melanoma immunotherapy. Their primary function is to protect mRNA from enzymatic degradation, facilitate cellular uptake, and ensure efficient release of mRNA transcripts in the cytosol of target cells. Following intramuscular (IM), IV, or subcutaneous administration, LNPs are internalized by APCs through endocytosis. After endosomal escape, the mRNA is released in the cytosol, where it undergoes translation into tumor-specific neoantigens [52]. These antigens are subsequently processed and presented on MHC Class I and II molecules, promoting activation of CD8^+^ cytotoxic and CD4^+^ helper T cells, respectively [24]. By this mechanism, LNP-mRNA vaccines enable durable antitumor responses through enhancement of neoantigen-specific T-cell expansion, cytokine-mediated recruitment, and potentiation of long-term immunologic memory [53].

Structurally, LNPs are commonly composed of four major elements:

1. Ionizable cationic lipid: promotes effective release of mRNA during target-cell endocytosis [54].
2. Phospholipid: bilayer formation for mRNA encapsulation [54].
3. Cholesterol: enhances membrane fluidity [54].
4. Polyethylene glycol-lipid conjugate: reduces aggregation and prolongs systemic circulation [54].

Progressive improvements in LNP formulation have led to three broadly described LNP generations [29]. First-generation LNPs use non-degradable lipids and exhibit modest transfection with relatively high toxicity [55,56,57]. Second-generation LNPs use biodegradable ester linkers to improve the safety profile and transfection efficacy [58]. Third-generation LNPs, such as those used in the COVID-19 mRNA vaccines, achieve the highest levels of transfection and can deliver longer mRNA constructs with lesser toxicity [59].

#### 4.2.2. Comparative Advantages

LNPs confer numerous advantageous benefits that have positioned them as the leading delivery vectors for mRNA cancer vaccines. LNPs provide protective encapsulation that shields mRNA from extracellular RNases, increasing stability, reducing off-target effects, and promoting more efficacious delivery of constructs to APCs [29,42,54,60]. This advantage becomes increasingly necessary as vaccine mRNA constructs are designed to encode more antigens.

LNPs substantially improve biodistribution and APC targeting relative to naked mRNA [60]. LNP-mediated mRNA delivery has been shown to elicit strong CD8^+^ cytotoxic T-cell responses and induce type I interferon signaling within DCs, contributing to the desirable pro-inflammatory, antitumor immune environment [61]. LNPs can be administered through a variety of routes, including IM, IV, intraduodenal, and subcutaneous administration [62]. Further, synthesis of LNPs entails a relatively simple, rapid process, easing the requirements for manufacturing and production at scale [62].

The modular structure of LNPs enables combination use with other compatible immunotherapies. For example, LNP-mRNA vaccines have been shown to enhance responses to ICIs in treatment-responsive melanoma patients, which may especially be relevant to these vulnerable populations [63]. Taken together, these properties highlight the substantial value of LNPs in advancing targeted, personalized melanoma vaccines.

#### 4.2.3. Limitations of Lipid Nanoparticle-Based mRNA Melanoma Vaccines

Current LNP technologies have notable limitations that constrain their therapeutic performance. One major challenge observed, especially with earlier generations, is off-target tissue accumulation, particularly within the liver. LNPs preferentially home to the hepatic tissues, which increases the risk of unintended immunogenicity or inflammatory tissue injury and reduces the proportion of mRNA delivered to target cells [29,64,65]. Further, although more theoretical in nature, concerns have been raised regarding rare instances of reverse transcription of delivered mRNA, potentially leading to genomic integration [29]. These concerns warrant further investigation and may underscore the need for safety monitoring when used in patients.

Despite improvements, transfection efficiency remains another important limitation. Even in the latest formulations, only a fraction of the administered LNPs successfully traffic to APCs and deliver their mRNA payload into the cytosol [64]. This shortcoming continues to direct attention towards increasing antigen output and immunogenic potency. 

Additional complexity arises when targeting ligands are incorporated into formulations to improve tissue specificity. Such modifications can complicate manufacturing, increase regulatory burden, and impede rapid production of mRNA vaccines. In response, Chen et al. demonstrated that a ligand-free LNP formulation can preferentially target lymph node tissue, even showing complete eradication of tumor cells in 40% of the murine melanoma cohort. Nonetheless, achieving efficient, selective delivery to APCs while minimizing off-target AEs remains a central obstacle. Current and investigational LNP generations aim to enhance transfection efficiency, focalize biodistribution, and minimize off-target AEs to optimize the immunogenicity of LNP-mRNA melanoma vaccines [29].

### 4.3. Dendritic Cell Vaccines and LNPs in Clinical Melanoma Trials

#### 4.3.1. Lipid Nanoparticle Vaccines—Completed Clinical Trials

In the KEYNOTE-942 Phase IIb trial, researchers evaluated mRNA-4157 (V940), a personalized LNP-based mRNA neoantigen vaccine, as adjuvant therapy for patients with completely resected stage IIIB–IV melanoma [37]. The vaccine was designed individually for each patient based on their tumor’s unique mutation profile and was given with pembrolizumab in 3-week cycles. Compared with pembrolizumab alone, the combination showed a clear improvement in recurrence-free survival, with a hazard ratio of 0.56 and almost half the recurrence or death events (22% vs. 40%). At 18 months, recurrence-free survival was 79% with combination therapy compared to 62% with pembrolizumab alone [37]. The safety profile was manageable, with mostly grade 1–2 toxicities and similar rates of immune-mediated events in both groups. These findings suggest that individualized LNP-mRNA vaccines can strengthen anti-PD-1 therapy in the adjuvant setting, likely by broadening endogenous and de novo T-cell responses against tumor-specific neoantigens.

In the KEYNOTE-603 Phase I trial, researchers evaluated mRNA-4157 (V940), an individualized LNP-based neoantigen mRNA vaccine designed to target up to 34 patient-specific tumor neoantigens in patients with completely resected stage II–IV melanoma [66]. Patients received 1 mg of mRNA-4157 either alone or in combination with pembrolizumab, and the study focused on safety and early immune responses. The vaccine showed a favorable safety profile, with all AEs limited to grade 1–3 and no dose-limiting or grade 4–5 toxicities. Mechanistically, mRNA-4157 induced strong neoantigen-specific immunity, generating both de novo T-cell responses and boosting pre-existing clones. The combination with pembrolizumab produced sustained expansion of cytotoxic CD4^+^ and CD8^+^ T cells that secreted IFN-γ and TNF-α in response to vaccine-encoded neoantigens, an effect that was not seen with anti-PD-1 therapy alone [66]. These findings provide early proof-of-concept that personalized LNP-mRNA vaccines can broaden antitumor T-cell responses in the adjuvant setting and meaningfully complement checkpoint inhibition.

These landmark clinical trials investigating LNP-based mRNA vaccines have demonstrated the potential of personalized neoantigen vaccination to enhance antitumor immunity in melanoma. Across both the KEYNOTE-942 Phase IIb and KEYNOTE-603 Phase I trials, the individualized mRNA-4157 (V940) vaccine, delivered using an LNP platform, was shown to be safe, well tolerated, and capable of generating robust neoantigen-specific T-cell responses. Together, these data support the idea that LNP-delivered personalized mRNA vaccines can work alongside checkpoint inhibition to strengthen mutation-targeted immune priming in the adjuvant setting. A detailed overview of completed clinical LNP-mRNA vaccine studies is provided in Table 1.

#### 4.3.2. Dendritic Cell Vaccines—Completed Clinical Trials

In a separate dendritic vaccine trial, patients with IIb-IV melanoma received an antigen-engineered DC vaccine designed to generate CD4^+^ and CD8^+^ responses against three shared melanoma antigens: (1) tyrosinase, (2) Melanoma Antigen Recognized by T cells 1 (MART-1), and (3) Melanoma-Associated Antigen A6 (MAGE-A6) [67]. After three doses of vaccination, patients were randomized to 1 month of observation or IFNα therapy. The DC vaccine was well tolerated, with high-grade toxicities linked to IFNα and not the vaccine itself. Median overall survival was 36 months, and median progression-free survival was 17.3 months. IFNα did not improve clinical outcomes (OS *p* = 0.54). 58% of patients developed vaccine-induced increased T-cell responses with increased circulating Tregs, MDSCs, and cytotoxic NK cells, but DC vaccine dose and IL-10 or IL-12 production did not correlate with benefit [67]. 

In a separate first-in-human randomized pilot phase II trial, HLA-A2 positive patients with advanced melanoma who were largely PD-1 resistant received a type 1 polarized monocyte-derived DC vaccine loaded with HLA-A2 restricted peptides from tumor blood vessel antigens (DLK1, EphA2, HBB, NRP1, RGS5, and TEM1) in combination with the tyrosine kinase inhibitor dasatinib [68]. Patients were assigned to one of two arms: Arm A received DC vaccination with dasatinib starting in week 5, and Arm B received DC vaccination with dasatinib starting in week 1. The combination was well tolerated with no treatment-related AEs above grade 3 and a toxicity profile consistent with dasatinib. Among 13 evaluable patients, 6 developed peripheral CD8^+^ T-cell responses against at least three vaccine peptides with evidence of epitope spreading to non-vaccine melanoma and vascular antigens; all six of these immunologic responders experienced clinical benefit, including four partial responses and two cases of stable disease, while most non-responders had disease progression. Patients treated on Arm B had higher immune and clinical response rates and improved survival compared with Arm A, with immune response rates of 66.7 versus 28.6 percent, objective response rates of 66.7 versus 0 percent, median overall survival of 19.1 versus 8.3 months (*p* = 0.0086), and median progression-free survival of 7.9 versus 2.2 months (*p* = 0.063) [68]. Mechanistically, vaccine benefit was linked to T-cell receptor convergence at baseline and during treatment, epitope spreading, and an inflamed tumor transcriptional profile with tertiary lymphoid structure-associated gene signatures, whereas non-responders had tumor signatures enriched for hypoxia, glycolysis, and cell cycle pathways. This trial highlights that DC-based vaccines can generate meaningful antitumor immunity even in PD-1-resistant melanoma, particularly when paired with agents that support T-cell priming and epitope spreading, like tyrosine kinase inhibitors. This trial also reinforces that patient-specific immune system factors, such as T-cell receptor convergence, MDSC levels, and a more overall inflamed tumor environment, are key determinants of vaccine success. 

A large prospective, randomized, double-blind phase IIb trial evaluating tumor-lysate DC vaccines further demonstrated the potential of DC-based immunotherapy in high-risk melanoma [69]. The study compared two related platforms: tumor lysate, particle-loaded DC (TLPLDC), which uses ex vivo-matured autologous DCs primed with tumor-lysate-loaded yeast cell wall particles, and tumor lysate, particle only (TLPO), an acellular formulation in which tumor-lysate-loaded particles are administered directly for in vivo DC priming. Across 187 patients with resected stage III/IV melanoma, both TLPLDC (without GM-CSF) and TLPO were well tolerated, with almost no related grade ≥3 AEs and predominantly mild injection-site reactions. Importantly, both vaccines were associated with meaningful survival benefits, with 36-month DFS of 55.4 percent for TLPLDC and 60.9 percent for TLPO compared with 27.2 percent in the placebo. Overall survival demonstrated a similar pattern, exceeding 93 percent in both vaccine groups. In contrast, pretreatment with GM-CSF, which is used to facilitate leukapheresis, produced a population of immature DCs and abrogated vaccine efficacy, with TLPLDC+G performing no better than placebo [69]. In exploratory subgroup analyses, patients treated with TLPO or TLPLDC in combination with checkpoint inhibitors demonstrated higher three-year disease-free survival rates than those in the placebo or TLPLDC+G groups, with more than 60 percent remaining disease-free. Together, these findings highlight that DC maturity is essential for effective priming, and they establish TLPO as an attractive next-generation option due to its similar efficacy, minimal toxicity, and substantial manufacturing advantages that eliminate the need for ex vivo DC harvest. A phase III study is now planned to validate TLPO in combination with contemporary adjuvant checkpoint blockade. 

A related ongoing study, NCT02678741, is evaluating the TLPLDC vaccine in patients with metastatic melanoma who are eligible for checkpoint inhibitor therapy [70]. This trial administers the TLPLDC vaccine, in which autologous DCs are matured ex vivo and primed with tumor-lysate-loaded yeast cell wall particles prior to reinfusion. The study is designed to assess the safety, feasibility, and immunologic activity of TLPLDC in combination with standard checkpoint blockade, with the goal of determining whether vaccine-induced priming can enhance response to PD-1-based therapy in the metastatic setting [70]. Although results have not yet been published, the trial represents an extension of prior TLPLDC work into advanced disease and may clarify the role of DC vaccination as an adjunct to checkpoint inhibitors in metastatic melanoma.

Another completed study, NCT03092453, evaluated a mature DC vaccine administered in combination with low-dose cyclophosphamide and sequential pembrolizumab in patients with advanced melanoma [71]. In this trial, autologous monocyte-derived DCs were matured ex vivo, pulsed with tumor antigens, and delivered intradermally as a priming strategy to enhance tumor-specific T-cell responses. Patients received cyclophosphamide 300 mg prior to the first vaccine dose to reduce regulatory T-cell activity and improve DC-mediated priming, followed by pembrolizumab 7–8 weeks after the final vaccine to sustain and expand vaccine-induced effector T cells. Although outcomes have not yet been published, this study reflects an important combination-strategy approach that integrates tumor antigen presentation, immune modulation, and checkpoint inhibition, aiming to improve antitumor immunity beyond what is achievable with PD-1 blockade alone. 

Preclinical investigations of DC-based vaccines have provided insight into how optimized antigen loading, DC maturation, and polarization strategies can shape antitumor immunity in melanoma. Across murine models, DC vaccines engineered with tumor lysates, melanoma-associated antigens, or peptide-loaded platforms consistently promoted expansion of cytotoxic CD8^+^ T cells, enhanced Th1-skewed cytokine production, and improved tumor control compared with unmodified or immature DC preparations. Collectively, these findings emphasize the importance of DC maturity, antigen presentation quality, and microenvironmental cues in determining the success of DC vaccine strategies. A detailed overview of completed DC vaccine studies is provided in Table 2. 

#### 4.3.3. Ongoing Clinical Trials

Several mRNA- and LNP-based vaccine trials remain active, reflecting continued expansion of personalized immunotherapy approaches in melanoma. The first, NCT06946225, is evaluating an individualized LNP-formulated mRNA neoantigen vaccine in patients with advanced melanoma who are receiving checkpoint inhibitor therapy [72]. The vaccine is designed based on each patient’s somatic mutation profile, with LNPs used to stabilize the mRNA transcript and enhance in vivo uptake by APCs. Although results are not yet available, this study may provide additional insight into how personalized LNP-mRNA vaccines function in the metastatic setting alongside immune checkpoint blockade.

Another key ongoing study is KEYNOTE-942 (NCT03897881), which serves as the pivotal, registration-intent trial building on earlier phase IIb data for mRNA-4157 (V940) [73]. This individualized LNP-based mRNA vaccine encodes up to 34 patient-specific neoantigens and is administered in combination with pembrolizumab in patients with resected high-risk melanoma. While initial data from the earlier phase of this program demonstrated a recurrence-free survival advantage, the full KEYNOTE-942 trial remains active, and its results will help determine whether LNP-delivered personalized mRNA vaccines become integrated into standard adjuvant melanoma care.

A third ongoing trial, NCT05533697, is investigating another LNP-formulated mRNA vaccine platform in patients with melanoma and other solid tumors [74]. This study focuses on evaluating safety, immunogenicity, and early signs of clinical activity, with the goal of determining whether LNP-mediated delivery of tumor-specific mRNA constructs can reliably elicit cytotoxic T-cell responses in diverse tumor contexts. As with other ongoing studies, results have not yet been published.

Together, these trials highlight the growing interest in personalized mRNA vaccines paired with advanced LNP delivery systems that enhance transcript stability, promote efficient antigen presentation, and support durable antitumor immune priming. A concise overview of all active studies is provided in Table 3.

### 4.4. Emerging Trends and Hybrid Strategies

#### 4.4.1. Advances in Dendritic Cell-Based Vaccine Engineering

Recent investigations have expanded the scope of dendritic cell (DC)-based mRNA vaccines by leveraging the distinct immunologic properties of specific DC subsets. Studies targeting Langerhans-type DCs, for example, have demonstrated that electroporation of mRNA encoding melanoma-associated antigens such as TRP-2 can enhance antigen-specific CD4^+^ and CD8^+^ T-cell responses, increase secretion of pro-inflammatory cytokines, and upregulate cytotoxic markers like CD107 [75].

DC fusion vaccines represent another emerging subcategory of DC vaccine technology. In both preclinical and clinical studies of melanoma, DC fusion cell vaccines yielded significant antitumor outcomes and facilitated the production of tumor-specific CTLs [42]. Primary techniques for producing DC fusion vaccines currently include electrofusion, viral fusion, and chemical fusion [42]. 

Combination DC-based strategies are also under active evaluation. Pairing DC vaccines with immune checkpoint inhibitors (ICIs) is showing promise in overcoming tumor microenvironment (TME)-mediated immunosuppression and reversing T-cell depletion, particularly in metastatic melanoma [76]. Early studies are likewise exploring synergies between DC vaccines and chimeric antigen receptor (CAR) T-cell therapy, wherein DCs are engineered to express tumor-associated antigens recognizable by CARs. This bidirectional interaction can enhance CAR T-cell activation, promote bystander T-cell priming, and reshape T-cell composition within the TME toward more durable antitumor responses [42]. 

While current mRNA vaccine strategies have centered on modulation of T-cell responses, recent evidence suggests that innate immune subsets, including NK cells, can indirectly contribute to antitumor immunity through exogenous stimulation of cytokine production and TME alteration. Small RNAs, such as microRNAs, have a role in immune response, aging, cellular senescence, and NK cell function and toxicity [77,78]. Additionally, small circular RNA vaccines have demonstrated efficacy in decreasing immunosuppression in melanoma models [79]. However, evidence linking small RNA to mRNA vaccine efficacy is limited, and further research is recommended to explore its potential effects [80]. 

#### 4.4.2. Innovations in LNP Design and mRNA Engineering

Parallel advances in lipid nanoparticle (LNP) technology are aimed at improving delivery efficiency, safety, and tissue specificity. One major limitation of current LNP platforms remains the difficulty of identifying promising candidates quickly enough for personalized vaccine development. High-throughput screening and sequencing-based methodologies may provide the means for accelerating the means for LNP candidate discovery and characterizing structure–function relationships relevant to melanoma [64]. 

Optimization of LNP chemistry and structure represents a rapidly expanding area of investigation. Incorporating biodegradable backbones, modifying the particle size, and altering lipid headgroup chemistry have all been associated with reduced hepatotoxicity and improved biodistribution of mRNA constructs [25]. Gene editing technologies have also entered the space. A recent study using CRISPR/Cas9-loaded, biodegradable LNPs targeted BRAF mutations and successfully reduced BRAF expression while inhibiting tumor growth, suggesting that multifunctional LNPs may serve dual roles as both vaccine vectors and therapeutic agents [81]. 

Hybrid nanoparticle platforms are additionally emerging to combine the immunologic benefits of biological systems with the structural advantages of synthetic carriers. Biomimetic nanoparticles, membrane-derived vesicles, and exosome-based systems are under investigation as alternative delivery vehicles capable of modulating the immunosuppressive melanoma microenvironment and enhancing DC priming. These carriers may be engineered to co-deliver mRNA alongside adjuvants such as TLR agonists or cytokines, thereby reversing “cold” TMEs and amplifying vaccine-induced T-cell responses. As physical delivery strategies evolve, electroporation, ultrasound-assisted loading, and microinjection techniques are being explored to further improve mRNA encapsulation efficiency and cellular uptake carriers [25,60,62,82].

#### 4.4.3. Neoantigen-Based Vaccines and Personalization

Neoantigen-based vaccination is advancing rapidly as one of the most promising avenues in melanoma immunotherapy. Neoadjuvant trials in melanoma, most notably those combining ipilimumab and nivolumab, have demonstrated longer event-free survival, reinforcing the rationale for integrating checkpoint blockade with upstream antigen-priming strategies, such as DC or LNP-based mRNA vaccines [83]. Peptide-based neoantigen vaccines have already shown the ability to expand antigen-specific T cells, promote epitope spreading, and generate more durable immune responses [84]. mRNA and DC-based platforms are now being adapted to encode similar personalized neoantigen repertoires, aiming to enhance potency while reducing manufacturing constraints [84,85]. 

Personalized neoantigen vaccines offer notable advantages over shared-antigen approaches, including reduced off-target toxicity and stronger, patient-specific immune activation [33]. While shared neoantigens derived from melanoma driver mutations hold promise for “off-the-shelf” formulations, they are relatively rare and often poorly presented by MHC molecules [86]. In contrast, private neoantigens, arising from the abundant passenger mutations characteristic of melanoma, are more immunogenic and uniquely suited for individualized vaccine design [86].

#### 4.4.4. Artificial Intelligence and Machine Learning

Advances in artificial intelligence (AI) and bioinformatics have enabled more sophisticated prediction of neoantigen binding, folding, and immunogenicity. AI algorithms utilize next-generation DNA and RNA sequencing data to accurately predict and prioritize neoantigen candidates that are most likely to be immunogenic [87]. Further, computational models of the tumor microenvironment, incorporating variables such as extracellular matrix density, abundance of infiltrating DCs, and T-cell composition, have begun to guide the rational design of vaccines optimized for specific tumor contexts. These tools may further support the development of mRNA-based platforms capable of tailoring antigen selection, adjuvant combination, and delivery properties to maximize therapeutic response [88]. 

One example of this approach is EVX 01, a personalized neoantigen cancer vaccine developed using an AI platform trained to identify and categorize patient-specific neoantigens in melanoma [89]. In the initial cohort of patients treated with EVX-01, all participants showed neoantigen-specific immune responses through CD4^+^ and CD8^+^ T cells after the priming phase [89]. The results of this study provide early clinical evidence of the supportive role of AI algorithms in (1) assisting with immunogenic target identification and candidate selection and (2) facilitating relatively fast, comprehensive development of highly personalized cancer vaccines for melanoma patients.

## 5. Conclusions

mRNA-based cancer vaccines represent one of the most exciting developments in melanoma immunotherapy, offering a level of personalization and immune precision that has not been achievable with prior treatment modalities. Across completed clinical and preclinical studies, both LNP-delivered mRNA vaccines and DC-based platforms have shown the ability to broaden neoantigen-specific T-cell responses, enhance antitumor immunity, and strengthen the effects of checkpoint inhibition. The encouraging outcomes seen with LNP-formulated vaccines such as mRNA-4157, paired with pembrolizumab, highlight the potential for individualized mRNA vaccines to meaningfully reduce recurrence risk in high-risk melanoma. Similarly, DC-based vaccines continue to demonstrate strong immunologic activity, especially when DC maturation and antigen loading are optimized to support durable T-cell priming.

Despite these advances, important challenges remain. Variability in tumor antigenicity, the immunosuppressive TME, and the complexities of mRNA delivery all continue to influence vaccine efficacy. Further work is needed to optimize delivery systems, improve DC and LNP targeting, enhance long-term immune memory, and integrate vaccines more seamlessly with existing ICI regimens. With the rise of personalized medicine and immunotherapy, further work is also needed to create personalized vaccines based on an individual’s antigens, cancer gene mutation maps, and overall genetic and epigenetic profiles. Ongoing clinical trials exploring next-generation LNPs, in vivo DC loading strategies, and combination approaches will help clarify how these platforms can be refined and brought into routine melanoma care. As our understanding of tumor neoantigens, RNA engineering, and delivery technologies continues to grow, mRNA vaccines hold tremendous promise for reshaping how we treat melanoma.

## Figures and Tables

**Table 1:** Overview of completed, landmark clinical trials evaluating personalized lipid nanoparticle (LNP)-delivered mRNA neoantigen vaccines for melanoma, including study phase, patient populations, interventions, and reported clinical and immune outcomes.

Trial ID and Phase	Title and Population	Intervention	Results
NCT03897881 (Phase IIb)	KEYNOTE-942 [37] Patients with high-risk resected melanoma (stage II–IV)	Individualized neoantigen therapy mRNA-4157 (V940) + pembrolizumab vs. pembrolizumab alone	Combination therapy improved recurrence-free survival (HR 0.561, 95% CI 0.309–1.017; *p* = 0.053), with fewer recurrence or death events (22% vs. 40%) and higher 18-month RFS (79% vs. 62%). Grade ≥ 3 treatment-related adverse events occurred in 25% vs. 18% of patients, with no vaccine-related grade 4–5 events. Immune-mediated adverse events occurred in 36% of both groups. Analysis demonstrated enhanced endogenous and de novo neoantigen-specific T-cell responses in the vaccine arm.
NCT03092453 (Phase II) Enrollment: 5	KEYNOTE-603 [66] Patients with resected cutaneous melanoma or NSCLC	Individualized neoantigen LNP-mRNA vaccine mRNA-4157 ± pembrolizumab	All participants experienced at least one treatment-emergent adverse event, all grade 1–3, with no dose-limiting or grade 4–5 toxicities. mRNA-4157 induced de novo and amplified neoantigen-specific CD4^+^ and CD8^+^ T-cell responses, including IFN-γ and TNF-α production and expansion of cytotoxic T-cell populations. Minimal neoantigen-specific activity was observed with pembrolizumab alone. Findings support mechanistic proof-of-concept for personalized LNP-mRNA vaccines in resected solid tumors.

**Table 2:** Summary of completed clinical trials evaluating dendritic cell-based vaccines for melanoma, including study phase, patient populations, vaccine platforms, and reported clinical and immune outcomes.

Trial ID and Phase	Title and Population	Intervention	Results
NCT01876212 (Phase II)	Dendritic Cell Vaccines + Dasatinib for Metastatic Melanoma HLA-2+ patients with metastatic melanoma	Combined DC vaccine targeting TBVAs +/− dastinib (tyrosine kinase inhibitor) [68] Arm A: delayed dastinib Arm B: immediate dasatinib	Six of 13 evaluable patients mounted CD8^+^ T-cell responses to at least three vaccine peptides with evidence of epitope spreading. Responders experienced four partial responses and two cases of stable disease, while non-responders had mostly progression. Immediate dasatinib produced higher immune response rates (66.7 vs. 28.6 percent), a higher objective response rate (66.7 vs. 0 percent), longer OS (19.1 vs. 8.3 months, *p* = 0.0086), and longer PFS (7.9 vs. 2.2 months, *p* = 0.063). Treatment was well tolerated with no grade ≥ 3 toxicities. Improved outcomes correlated with TCR convergence, epitope spreading, and development of an inflamed, TLS-associated tumor signature.
NCT03092453 (Phase II)	Dendritic Cell Vaccination in Patients with Advanced Melanoma Stage III and IV melanoma patients	DC vaccine; cyclophosphamide 300 mg given before the first dose; pembrolizumab administered 7–8 weeks after the final DC dose [71]	Serious adverse events included tumor pain (1/5) and palliative mass resection (1/5). Other reported symptoms included tachycardia, abdominal pain, and nausea in 60 percent of patients. No additional published clinical outcomes are available.
NCT02678741 (Phase I + II)	Multi-center Phase I/IIa Trial of an Autologous Tumor Lysate (TL) + Yeast Cell Wall Particles (YCWP) + Dendritic Cells (DC) Vaccine in Addition to Standard of Care Checkpoint Inhibitor of Choice in Metastatic Melanoma Patients With Measurable Disease. Metastatic melanoma patients eligible for CPI (checkpoint inhibitor) therapy	Tumor Lysate, Particle-Loaded, Dendritic Cell Vaccine (TLPLDC) Vaccine [70]	Sixteen of 26 patients received at least one vaccine dose. Among evaluable patients, 12.5 percent showed no clinical response, and 62.5 percent had progressive disease following vaccination. No serious adverse events were reported. Mild-to-moderate AEs included syncope, nausea, and headache. No additional published clinical outcomes available.
NCT01622933 (Phase I)	Multiple Antigen-Engineered DC Vaccine for Melanoma Recurrent and inoperable stage III, IV, or metastatic melanoma previously treated with any form of therapy	DC Vaccine + IFN vs. AdVTMM2/DC Vaccine only (contains tyrosinase, MART-1 and MAGE-A6) [67]	Among 24 patients with measurable disease, the DC vaccine produced 2 partial responses and 8 cases of stable disease; 4 of 11 NED patients remained disease-free at a median 3-year follow-up. Median OS was 36 months, and median PFS was 17.3 months, with no added benefit from IFNα (OS *p* = 0.54; PFS *p* = 0.43). Antigen-specific CD4^+^ and CD8^+^ T-cell responses increased in 18/31 evaluable patients. DC vaccine dose, phenotype, and IL-12p70 or IL-10 secretion were not predictive of outcome. Improved clinical outcomes correlated with lower MDSC levels and specific effector-memory CD4^+^/CD8^+^ subsets and CD56lowCD16^+^ NK cell frequencies.
NCT02301611 (Phase IIb)	Phase IIB TL + YCWP + DC in Melanoma Stage III and IV melanoma patients	TLPLDC vs. TLPO vaccine ± G-CSF pretreatment [69]	At 36 months, TLPLDC and TLPO demonstrated higher disease-free and overall survival compared with placebo, with DFS of 55.4 percent and 60.9 percent, and OS of 93.6 percent and 94.6 percent, respectively. In contrast, TLPLDC, formulated after G-CSF pretreatment, which produced immature, IL-10-skewed dendritic cells, showed no clinical benefit and performed similarly to placebo. DFS was 27.2% (placebo), 55.4% (TLPLDC), 22.9% (TLPLDC+G), and 60.9% (TLPO) (*p* < 0.001). OS was 62.5% (placebo), 93.6% (TLPLDC), 57.7% (TLPLDC+G), and 94.6% (TLPO) (*p* = 0.002). The TLPO vaccine showed comparable efficacy to TLPLDC with substantial manufacturing advantages, supporting its advancement to phase III evaluation.

**Table 3:** Overview of ongoing clinical trials evaluating personalized mRNA and lipid nanoparticle (LNP) vaccine platforms for melanoma, including trial phase, target populations, estimated enrollment, and investigational treatment strategies.

Trial ID	Title and Estimated Enrollment	Population	Estimated Enrollment	Intervention	Summary
NCT06946225 (Phase I)	ACTengine^®^ IMA203 Combined With mRNA-4203 [72]	HLA-A02:01 positive patients with previously treated or unresectable metastatic cutaneous melanoma or synovial sarcoma	15	Single infusion of IMA203 followed by 10 days of IL-2 after lymphodepleting chemotherapy; mRNA-4203 administered beginning on day 15 for up to 12 cycles (28-day cycles).	Noncomparative study evaluating safety, tolerability, and antitumor activity of the IMA203 + mRNA-4203 combination in HLA-A02:01-positive melanoma.
NCT03897881 (Phase II)	An Efficacy Study of Adjuvant Treatment With the Personalized Cancer Vaccine mRNA-4157 and Pembrolizumab in Participants With High-Risk Melanoma (KEYNOTE-942) [73]	Patients with completely resected cutaneous melanoma at high risk for recurrence	267	mRNA-4157 + Pembrolizumab	Comparative study evaluating if mRNA-4157 and pembrolizumab adjuvant improves recurrence-free survival (RFS) compared to pembrolizumab alone.
NCT05533697 (Phase I + II)	Study of mRNA-4359 Administered Alone and in Combination With Immune Checkpoint Blockade in Participants With Advanced Solid Tumors [74]	Patients with advanced or metastatic solid tumors, including cutaneous melanoma, NSCLC, non-muscle invasive bladder cancer, head and neck squamous cell carcinoma, MSS colorectal cancer, basal cell carcinoma, and triple-negative breast cancer.	361	mRNA-4359 administered alone or in combination with pembrolizumab, or with ipilimumab plus nivolumab.	Study assessing the safety and tolerability of mRNA-4359 administered alone and in combination with immune checkpoint inhibitors.

## Data Availability

No new data was created or analyzed in this study. Data sharing is not applicable to this article.

## Abbreviations

The following abbreviations are used in this manuscript:

AE	adverse event
AI	artificial intelligence
APC	antigen-presenting cell
CAR	chimeric antigen receptor
cDC	conventional dendritic cell
COVID-19	coronavirus disease 2019
CRISPR	Clustered Regularly Interspaced Short Palindromic Repeats
CTL	cytotoxic T lymphocytes
CTLA-4	cytotoxic T-lymphocyte-associated protein 4
DC	dendritic cell
DNA	deoxyribonucleic acid
DNS	dysplastic nevus syndrome
FAMMM	familial atypical multiple mole and melanoma syndrome
G-CSF	granulocyte colony-stimulating factor
GM-CSF	granulocyte-macrophage colony-stimulating factor
HLA	human leukocyte antigen
HSV-1	herpes simplex virus-1
ICI	immune checkpoint inhibitor
IFN	interferon
IL	interleukin
IM	intramuscular
IV	intravenous
JAK/STAT	Janus kinase/Signal Transducer and Activator of Transcription
LNP	lipid nanoparticle
MAGE-A6	melanoma-associated antigen A6
MAPK	mitogen-activated protein kinase
MART-1	Melanoma Antigen Recognized by T cells 1
MC1R	melanocortin-1 receptor
MDSC	myeloid-derived suppressor cells
MHC	major histocompatibility complex
MITF	microopthalmia-associated transcription factor
MoDC	monocyte-derived dendritic cell
mRNA	messenger ribonucleic acid
NF-κB	nuclear factor-κB
NK	natural killer
pDC	plasmacytoid dendritic cell
PD-1	programmed death-1
PD-L1	programmed death-ligand 1
TAA	tumor-associated antigen
TBVA	tumor blood vessel antigens
Tfh	T follicular helper cells
TLR	toll-like receptor
TLPLDC	Tumor Lysate, Particle-Loaded, Dendritic Cell Vaccine
TLPO	tumor lysate, particle only
TME	tumor microenvironment
TNF-α	tumor necrosis factor-α
T-VEC	oncolytic viral therapy
TSA	tumor-specific antigen
UV	ultraviolet
